# Portal vein thrombosis as a thrombotic complication of COVID-19 mRNA vaccine: A case report and literature review

**DOI:** 10.1016/j.idcr.2022.e01582

**Published:** 2022-07-26

**Authors:** Prakriti Singh Shrestha, Angela Ishak, Arun R. Napit, Sarosh Sarwar, Niraj Rai, Zoha Nizami, Niharika Bheemisetty, Prashanth Jayaraj, Amardeep Shrestha, Ivan D. Rodriguez

**Affiliations:** a Department of Research and Academic Affairs, Larkin Community Hospital, South Miami, FL, USA; b Valley Health and Research Center, Srijananagar, Bhaktapur, Nepal; c Fazaia Medical College, Islamabad, Pakistan; d University of Health Sciences, Lahore, Pakistan; e Department of Family Medicine, Larkin Community Hospital, South Miami, FL, USA; f Family Medical Clinic, O’Fallon, IL, USA

**Keywords:** COVID-19 vaccine, Portal vein thrombosis, VITT

## Abstract

Thrombosis following COVID-19 vaccination commonly occurs with vector-based vaccines. The proposed mechanism is vaccine-induced thrombotic thrombocytopenia (VITT), with thrombocytopenia as principal manifestation. We present a 51-year-old male who came with isolated portal vein thrombosis (PVT) one day after Moderna vaccination, without associated thrombocytopenia, challenging VITT as being the only patho-mechanism. Further exploration of these possible alternative mechanisms is needed for COVID-19 vaccine-related thrombotic complications.

## Background

COVID-19 vaccine roll-out started in December 2020 after novel coronavirus first emerged in December 2019 in Wuhan, China, devastating the world with a new pandemic [1]. Since the enrollment of the COVID-19 vaccines, there have been reports of several adverse events associated with these vaccines such as symptoms of injection-site soreness, headache, flu-like symptoms, and fever, mostly observed with Pfizer, Moderna, and AstraZeneca [2]. Thrombosis was one of the most concerning adverse effects of COVID-19 vaccines. Most of these cases were reported exclusively after vector-based vaccines (AstraZeneca and Janssen vaccine) and less with mRNA vaccines (Pfizer-BioNTech or Moderna) [3]. Nevertheless, these thrombotic events were pathologically related to vaccine induced thrombotic thrombocytopenia (VITT) syndrome where thrombocytopenia is one of the diagnostic manifestations [4]. These thrombosis were seen at various sites and organs including cerebral venous sinuses, portal, and splanchnic circulation [5].

This case report describes an isolated portal venous thrombosis which occurred within a day of COVID-19 vaccination, not associated with thrombocytopenia, and further, was associated with one of the mRNA-based vaccine, Moderna. To our knowledge, this is the only case report with isolated portal venous thrombosis related to mRNA-based vaccine. This case also challenges the belief of VITT as a potential patho-mechanism causing thrombosis related to COVID-19 vaccine and questions whether there are other mechanisms that could be in play.

## Case presentation

A 51-year-old male with a past medical history of diabetes, obesity, hyperlipidemia, hypertension, and coronary artery disease status post coronary angioplasty in remote past, presented to the emergency department with complaints of severe upper abdominal pain that started one day after receiving his first dose of Moderna vaccine. He denied fever, chills, nausea, vomiting, chest pain, night sweats, or weight loss. He did not have any significant history of coagulopathy in the past. He was compliant with his medications which included insulin, metformin, amlodipine, benazepril, rosuvastatin, and citalopram. He did not smoke, consume alcohol, or used any illicit drugs. He had no family history of clotting disorder.

On physical examination, he was normotensive with a normal heart rate and was afebrile. He was in distress with pain and had epigastric tenderness on abdominal palpation. The rest of the examination was within normal limits. Complete blood count showed no abnormalities with a baseline platelet count of 144 × 10^3^/μl. The comprehensive metabolic profile was within normal limits. Liver function tests were normal (total bilirubin 0.5 mg/dL, alanine transaminase (ALT) 31 IU/L, aspartate aminotransferase (AST) 34 IU/L, alkaline phosphatase (ALP) 64 IU/L). Amylase and lipase levels were normal at 42 and 23 IU/L, respectively. The coagulation profile and d-dimer (< 150 ng/mL) were normal. Inflammatory markers (ferritin 239 ng/mL) were unremarkable. Alpha-fetoprotein (AFP) level and alpha-1 antitrypsin antibodies were normal, and ANA, hepatitis B, and C tests came back negative.

**Fig. 1:**
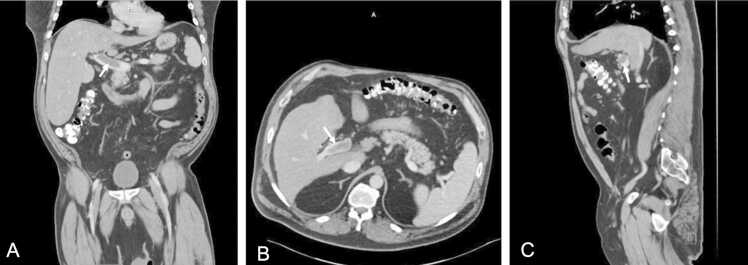
CT abdomen and pelvis with contrast at the emergency department a) coronal, b) transverse and c) sagittal: evidence of thrombus in the portal vein and superior mesenteric vein (white arrow).

**Fig. 2:**
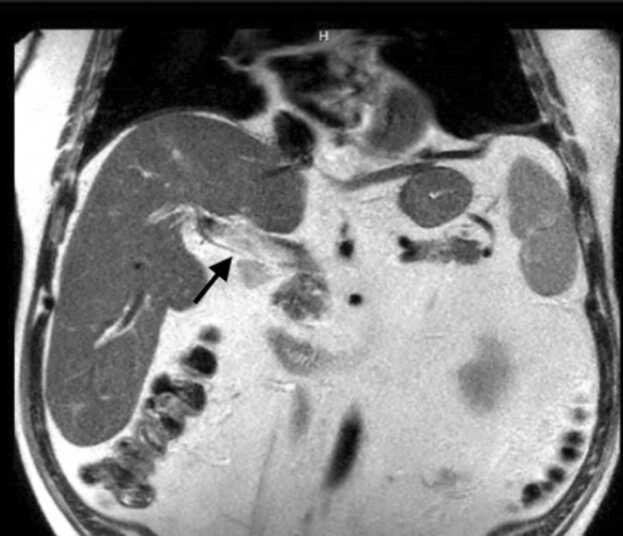
MRI of the abdomen and pelvis without contrast showing evidence of thrombus portal vein (black arrow).

Computed tomography (CT) of abdomen and pelvis at the emergency department revealed evidence of thrombus in the portal vein and superior mesenteric vein along with a somewhat nodular contour of liver suggesting underlying chronic liver parenchymal disease [Fig. 1**]**. This was followed by a magnetic resonance imaging (MRI) of the abdomen which revealed a similar nonocclusive thrombus within the main portal vein extending into the proximal aspects of the right and left portal veins [Fig. 2]. The liver showed diffuse hepatic steatosis.

On further investigation, homocysteine level was normal, Beta-2 glycoprotein I immunoglobulin G (IgG), and immunoglobulin M (IgM) was negative. Protein C and S activity were within normal limits. Antithrombin activity revealed low activity at 50 % initially, but a repeat was negative. Anticardiolipin Antibodies IgG and IgM were negative. Activated protein C resistance testing was negative for Factor V Leiden. Prothrombin gene mutation was negative. Table 1 shows the trend of the patient’s platelet count from previous levels to the day of hospitalization and subsequent levels. There were no clinical signs and symptoms of active COVID-19 infection at that time and hence COVID PCR testing was not performed.

**Table 1:** Trends of platelet count is tabulated below comparing it with his previous values.

**Day**	4/25/2019	11/28/2021	2/1/2021	3/10/2021	6/28/2021 (ED visit)	6/29/2021	6/30/2021	7/5/2021	7/14/2021
**Platelet count (10**^**3**^**//μl)**	134	121	133	130	144	121	124	153	135

**Fig. 3:**
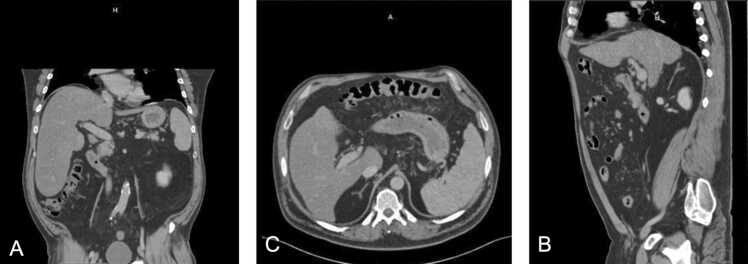
Repeat abdominal CT scan with contrast on follow-up 3 month later (portal vein thrombosis has resolved) a) coronal, b) transverse and c) sagittal.

The patient was admitted and was started on heparin therapy during the hospital stay. He was discharged on apixaban 10 mg twice daily. He continued to follow up with hematologist. A follow up CT scan 3 months later revealed resolution of previously found thrombosis [Fig. 3**]**. His abdominal pain also continued to improve and is currently maintained on apixaban 5 mg twice daily.

## Discussion

Portal vein thrombosis (PVT) is a rarely experienced clinical entity involving thrombosis within the portal vein trunks and intrahepatic portal branches [6]. It is commonly seen with cirrhosis of the liver causing portal hypertension [6]. Some of the other etiologies that require a careful evaluation are underlying congenital or acquired coagulation disorders such as Factor V Leiden mutation, prothrombin gene mutation, antiphospholipid syndrome, underlying neoplastic disorders, or infectious and autoimmune disorders [7]. With the emergence of the COVID-19 vaccine, there has been reported cases of PVT after adenovirus vector COVID-19 vaccine administration due to VITT [8], [9].

Laboratory findings for PVT are nonspecific and non-diagnostic[10] as seen in our patient. Ultrasonography is most often utilized to diagnose PVT, which presents as hypo- or iso-echoic shadow within the dilated vein [11]. Abdominal CT and computed tomographic angiography (CTA) are the most widely used tools for diagnosing PVT, as well as to rule out malignancy causing PVT [10], [11]. MRI is the first-line diagnostic tool for PVT due to its high sensitivity [11]. The primary aim of the treatment of PVT is to intercept the progression of thrombus formation and reopen the blockage [6]. Anticoagulants are the first-line treatment for the management of PVT. The commonly utilized anticoagulants are heparins (unfractionated heparin and low molecular weight heparin) vitamin K antagonist, and direct oral anticoagulants, including factor Xa inhibitors (Apixaban, Rivaroxaban) and factor IIa inhibitors (Argatroban, Dabigatran) [6], [7], [12]. Depending upon the severity of the obstruction, thrombolysis, thrombectomy, and TIPS are also performed [6], [7], [11]. Additionally, intravenous immunoglobulin has been used as a therapeutic agent in PVT due to VITT after vaccination with AstraZeneca for COVID [8], [13].

VITT has been related to most of these thrombotic events following COVID-19 vaccination. It is an autoimmune phenomenon typically occurring 5–42 days after the vaccination and is characterized by thrombocytopenia, elevated d-dimer, increased antiplatelet factor 4 (PF4) antibodies, along with thrombosis [14]. Once there is entry of vaccine particle, it interacts with PF4 antibodies, a molecule stored in platelet alpha granules, which gets released during platelet activation [14]. Further, these PF4 molecules opsonize the particles, facilitating binding of anti-PF4 antibodies from preformed B cells. In VITT, there is an increased rise in the titer of antiplatelet factor 4 antibodies that binds to the platelets Fc gamma receptor leading to platelet activation and release of procoagulant particles [14]. Hence, VITT will involve consumptive coagulopathy with thrombocytopenia, hypofibrinogenemia and elevated d-dimer. This closely resembles heparin induced thrombocytopenia (HIT) [15]. The antibodies usually develop between 4 and 16 days following the antigen activation [16]. Hence, there is a lag of thrombotic complications to develop following the inciting event.

It is not understood as of now on the mechanism of antibodies production. In vector-based vaccine, adenovirus attached to the thrombocytes likely lead to their activation [15]. Another hypothesis is that the vaccine's genetic material (DNA or RNA) may have contributed to the production of reactive antibodies supporting physiology behind Moderna vaccine [17]. The incidence and pathogenesis of venous thrombosis after mRNA COVID-19 vaccines remain unknown and appears to be rare [5]. Fan et al. presented three cases and Dias et al. reported two cases of cerebrovascular vein thrombosis (CVT) in patients who received Pfizer vaccine [18], [19]. Causality has not been proven.

VITT is by far the most sought mechanism for vaccine-induced thrombosis. However, our case did not meet the diagnostic criteria for VITT. In this case, thrombosis occurred within a day of vaccination which is not typical for an autoimmune phenomenon. Several etiological factors including hypercoagulability revealed no specific findings. Though COVID-19 testing was not performed on the day of admission or prior to that, he was not having symptoms pertaining to COVID-19 infection in the recent past. Hence, we hypothesize that vaccine might be causing thrombosis. VITT is a consideration in this setting, however, our patient did not have any worsening of his platelet count during the event as depicted in our table. Graça et al. present a similar case but related to the AstraZeneca vaccine [20]. Lack of thrombocytopenia and occurrence of the event within a day after COVID vaccination in both of these cases question whether there are other mechanisms that could play.

## Conclusion

Portal vein thrombosis and other thrombotic complications are rare adverse events of COVID-19 mRNA vaccine. VITT is noted to be the only mechanism known to date that has been associated with COVID vaccination. Whether there is indeed some other mechanism, as depicted in our case, needs further investigation. We also want to emphasize that these complications, though rare, needs appropriate workup, evaluation, and management. Regardless of these or other adverse events, we certainly do believe that the benefits of vaccine outweigh overall risk.

## CRediT authorship contribution statement

AS and PSS were involved in the patient care and conceptualized the case report. AS, PSS, AI, ARN, SS, NR, ZN, and NB were involved in drafting the manuscript. AS, PSS, AI and PJ were involved in editing the final version of the manuscript. AI and PSS prepared and edited figures. AS and IDR supervised and critically revised the project. All authors read the final version and approved the manuscript.

## Conflict of Interest

None to disclose.
